# Extracellular Vesicles in Physiology, Pathology, and Therapy of the Immune and Central Nervous System, with Focus on Extracellular Vesicles Derived from Mesenchymal Stem Cells as Therapeutic Tools

**DOI:** 10.3389/fncel.2016.00109

**Published:** 2016-05-02

**Authors:** Sylwia Koniusz, Anna Andrzejewska, Maurizio Muraca, Amit K. Srivastava, Miroslaw Janowski, Barbara Lukomska

**Affiliations:** ^1^NeuroRepair Department, Mossakowski Medical Research Centre, Polish Academy of SciencesWarsaw, Poland; ^2^Department of Women’s and Children’s Health, University of PaduaPadua, Italy; ^3^Russel H. Morgan Department of Radiology and Radiological Science, Division of MR Research, The Johns Hopkins University School of Medicine, BaltimoreMD, USA

**Keywords:** extracellular vesicles, mesenchymal stromal cells, CNS, neurological diseases, biomarkers, transplantation

## Abstract

Extracellular vesicles (EVs) are membrane-surrounded structures released by most cell types. They are characterized by a specific set of proteins, lipids and nucleic acids. EVs have been recognized as potent vehicles of intercellular communication to transmit biological signals between cells. In addition, pathophysiological roles of EVs in conditions like cancer, infectious diseases and neurodegenerative disorders are well established. In recent years focus has been shifted on therapeutic use of stem cell derived-EVs. Use of stem cell derived-EVs present distinct advantage over the whole stem cells as EVs do not replicate and after intravenous administration, they are less likely to trap inside the lungs. From the therapeutic perspective, the most promising cellular sources of EVs are mesenchymal stem cells (MSCs), which are easy to obtain and maintain. Therapeutic activity of MSCs has been shown in numerous animal models and the beneficial paracrine effect of MSCs may be mediated by EVs. The various components of MSC derived-EVs such as proteins, lipids, and RNA might play a specific therapeutic role. In this review, we characterize the role of EVs in immune and central nervous system (CNS); present evidences for defective signaling of these vesicles in neurodegeneration and therapeutic role of EVs in CNS.

## Introduction

Mesenchymal stem/stromal cells (MSCs) are of great interest in regenerative therapy for tissues damaged by various pathological conditions. The chief therapeutic attributes of MSCs are their ability to migrate into injured sites (Kraitchman et al., 2005; Kim et al., 2015), promote functional recovery and modulate immune responses. Although the process of MSC homing is not very effective and there were various strategies attempted to enhance it. The engineering of MSC facilitates reaching target organs (Nowakowski et al., 2016). Another approaches are based on more direct routes of cell delivery, which, however, are slightly more invasive. After finding conditions determining the safety of intra-arterial delivery (Janowski et al., 2013; Cui et al., 2015), this route has been found effective in animal model of stroke (Toyoshima et al., 2015). The intrathecal route is even less invasive and was also shown effective (Lim et al., 2011). The intracerebral route is more invasive and is going to be used rather as an addition to neurosurgical treatment such as evacuation of hematoma (Zhang Q. et al., 2015). In general, it was thought that the close proximity of transplanted MSCs is pivotal in achievement of substantial therapeutic effect, as the cells could act through various mechanisms such as direct cell-to-cell contact and secreted factors. However, an extensive meta-analysis of preclinical results of intravenous application of stem cell revealed that there is a good correlation between a dose of infused cells and therapeutic effect, however, such correlation does not exist between the outcome and number of cells that engrafted within the disordered brain area (Janowski et al., 2010). It indicates that, there are substantial therapeutic mechanisms that are not directly related to the presence of cells within the injury site. Additional, accumulating evidence over the past few years supports the notion that the predominant mechanism by which MSCs act in tissue repair is mainly related to their paracrine/secretory effect. Indeed, MSCs provide microenvironment with a multitude of trophic signals including growth factors and cytokines. It is likely that in parallel to soluble factors, MSCs release EVs that contribute to the reparative process by intercellular cross talk communication. The biological relevance of extracellular vesicles (EVs) that mirrors parental cells has been established in different experimental settings. Recent discoveries suggest that they have similar protective properties as their cellular counterparts to condition and reprogram the surrounding microenvironment influencing a variety of endogenous responses in particular in injured tissues. It has been shown that EVs can affect other cells via transfer of genetic cargo, transfer of receptors and ultimately initiating pathways (Momen-Heravi et al., 2013). They are able to modify cell fate, function and plasticity. Recent data indicate that EVs have the capacity to modulate immune response and facilitate tissue regeneration. Therefore the use of them may represent an interesting alternative therapy for various diseases compared to a cell-based approach. EVs released by MSCs are heterogeneous population that differs in size and biogenesis. They contain proteins, bioactive lipids and nucleic acids that can mediate various signaling functions contributing to homeostasis. Transfer of these molecules to neighboring cells promotes cell-to-cell communication and modifies the activity of target cells. In the central nervous system (CNS) probably more than in other organs such communication between neurons and glial cells is very crucial in physiological conditions. There is also strong evidence that EVs play a role in learning and memory (Smalheiser, 2007). Moreover, the biomolecules delivered by these structures may support and protect neurons, remove debris and infectious agents as well as control inflammation in pathological situation. They are involved in removal of misfolded proteins, harmful cell metabolic products and viral particles (Inal et al., 2012; Ohno et al., 2013). The recent literature implicates that microvesicles have the potential to transfer a collection of biomolecules between cells locally or over long distance through the blood or other biological fluids (Baglio et al., 2012; Frühbeis et al., 2013; Raposo and Stoorvogel, 2013). The circulation half-life of EVs in blood is approximately 2 min (Takahashi et al., 2013; Saunderson et al., 2014) but they have been detected in lungs, liver, spleen, and pancreas 48 h after systemic injection (Wiklander et al., 2015). This opens novel therapeutic perspectives aimed at the development of cell-free strategies based on the use of MSCs secretome as a potentially more advantageous alternative to cell-based therapy approaches.

In this review we summarize role of EVs in pathophysiology of different neurological and immunological conations, properties and functions of EVs derived from MSCs and their potential therapeutic role in neurological disorders.

## Types and Production of EVs

**FIGURE 1:**
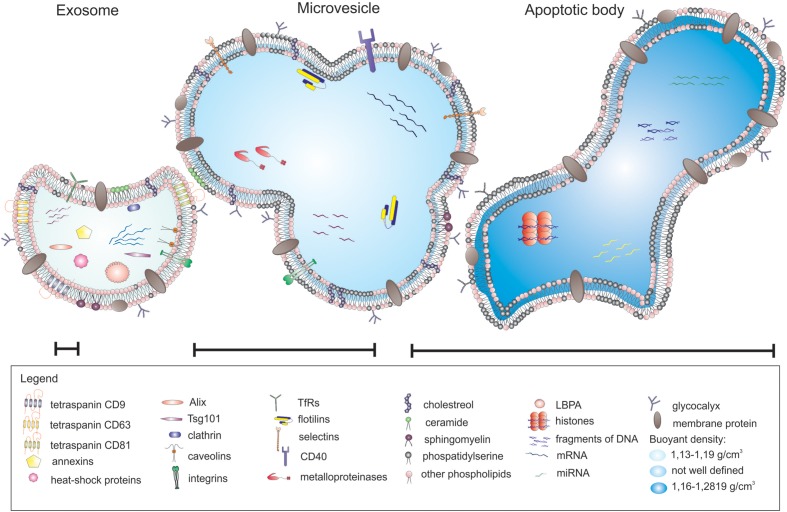
**Different types of extracellular vesicles (EVs) released from parental cells**.

Extracellular vesicles can be categorized into three main classes based on their mode of origin: exosomes, shedding microvesicles and apoptotic bodies (**Figure 1**). In broad terms there are three types of EVs, however, in the literature the nomenclature is inconsistent and the term microvesicles is often used as an umbrella term to encompass exosomes and shedding microvesicles (Lai et al., 2015).

Exosomes represent a specific subtype of secreted vesicles. They are presently the best-characterized species of EVs. Exosomes arise in the endocytic pathway and are released by exocytosis through a mechanism dependent on cytoskeleton activation regulated by p53 protein but independently of cell calcium influx (Tetta et al., 2011; Biancone et al., 2012). They are small spherical vesicles with a size of 30–120 nm, cup shaped, limited by lipid bilayer, and constituted a rather homogenous population. Exosomes are derived from late endocytic compartments, known as multivesicular bodies (MVBs). They are produced by inward invagination of endosomal membranes to form MVBs, which subsequently fuse with the plasma membrane and release their intraluminal vesicles as exosomes to the extracellular milieu (Lai and Breakefield, 2012). As endocytosis is most active at specific unique micro domains in the plasma membrane called lipid rafts, exosomes have membranes enriched in elements of lipid rafts such as GM1 gangliosides and transferrin receptors (Tan et al., 2013). They are rich in annexins, tetraspanins (CD63, CD81, and CD9) and heat shock proteins (Hsp60, Hsp70, and Hsp90), expose clathrin, calveolins as well as endosome-specific proteins such as Alix and Tsg101 and cell-type specific proteins (Biancone et al., 2012; Frühbeis et al., 2013; Sabin and Kikyo, 2014). Exosomes carry characteristic lipids and contain cholesterol, ceramide, sphingomyelin, and phosphatidylserine (Subra et al., 2007). As mentioned above, nucleic acids such as mRNA and miRNA are also present in exosomes (Mathivanan et al., 2010).

Shedding vesicles known as ectosomes or microvesicles are another class of EVs. As the name implies, they are shed directly from the plasma membrane of the cell. Microvesicles are heterogeneous in size, ranging from 100 nm to 1 μm. Their release is initiated by outward budding from the membrane surface followed by a fission event similar to the abscission step observed in cytokinesis (Turturici et al., 2014). Shedding of vesicles is physiological phenomenon that accompanied cell activation and growth. Their detachment from small cytoplasmic protrusions depends on calcium influx, calpain, scramblase, floppase, and cytoskeleton reorganization (Cocucci et al., 2009; Tetta et al., 2011). Calcium ions are responsible for the changes in asymetric phospholipid distribution of the plasma membrane that lead to the formation of shedding vesicles (Biancone et al., 2012). The release of microvesicles occurs from all types of cells in resting state, or upon activation by soluble factors or oxidative stress, hypoxia or shear stress. However, the main function of shedding vesicles is signaling through specific interaction with target cells and the transfer of genetic information (mRNA). Microvesicles influence the behavior of target cells in multiple ways, such as signaling complexes by direct stimulation, transferring receptors between cells or delivering proteins. Microvesicles may transmit miRNA to neighboring cells that can alter the expression of genes in these cells. The content of microvesicles differs to some extent from that of exosomes. Shedding vesicles lack proteins of the endocytic pathway but they expose high amounts of phosphatidylserine, contain protein associated with lipid rafts such as integrins and flotillins and are enriched in cholesterol, sphingomyelin, and ceramide (Mathivanan et al., 2010). Although tetraspanins are considered as unique markers for exosomes, they can be expressed in shedding microvesicles in some cases.

Apoptotic bodies represent another type of EVs. Although they resemble microvesicles they can be distinguished by their large size and irregular shape (György et al., 2011). In contrast to exosomes and microvesicles that derive from healthy cells, apoptotic bodies are released during the apoptosis. They are formed during the late stage of apoptosis and contain nuclear material, cellular organelles and membrane contents. They express phosphatidylserine on their surface and have a permeable membrane (Turturici et al., 2014). Apoptotic bodies tend to elicit an anti-inflammatory or tolerogenic response when taken up by neighboring cells (Lai et al., 2015; **Table 1**).

**Table 1:** The properties of different types of extracellular vesicles (EVs).

Property	Exosomes	Microvesicles	Apoptotic bodies
Size	30–120 nm	100–1000 nm	50–4000 nm
Morphology	Homogenous cup-shape	Heterogeneous irregular	Heterogeneous irregular
Buoyant density	1,13–1,19 g/cm^3^	Not well defined	1,16–1,28 g/cm^3^
Origin	Endosomal	Plasma membrane	Apoptotic cells
Proteins	CD63, CD81, CD9, annexins, heat-shock proteins, Alix, Tsg101, clathrin, caveolins, integrins, TfRs	Integrins, flotillins, selectins, CD40, metalloproteinases	Histones
Lipids	Lysobisphosphatidic acid, cholesterol, ceramide, sphingomyelin and low concentration of phosphatidylserine	High amount of cholesterol, sphingomyelin, ceramide, high concentration of phosphatidylserine	High concentration of phosphatidylserine
Nucleic acids	mRNA and miRNA	mRNA and miRNA	mRNA, miRNA, fragments of DNA


## Proteomic Analysis of MSC-EVs

Extracellular vesicles are composed of various molecules including proteins, lipids, and nucleic acids. Secreted proteins participate in intercellular communication and play a role in cell signaling, differentiation, cell adhesion, angiogenesis, and apoptosis. A variety of cytokines, chemokines, growth factors, extracellular matrix (ECM) proteins and remodeling enzymes have been identified in MSCs derived from different sources and their secretomes (Kupcova Skalnikova, 2013).

The initial proteomic characterization of EVs secreted from MSCs was published by Kim et al. (2012) and Lai et al. (2012). Kim et al. (2012) using mass spectrometry profiled the proteome of microvesicles (in size from 50 to 200 nm) which were harvested by ultracentrifugation from human bone marrow MSCs and identified 730 proteins. Lai et al. (2012) distinguished 857 proteins with the same technique in exosomes isolated from human ESC-derived mesenchymal stem cells (MSCs; huES9.E1) line by high performance liquid chromatography. In these two articles we can find a common subset of 315 proteins (**Supplementary Tables S1** and **S2**). Among the sets of proteins, in addition to cytoplasmic proteins a remarkable number of membrane proteins have been found. The proteins located in the plasma membrane and cytoplasm are more commonly sorted into EVs compared with the proteins in the nucleus and mitochondria of different cell types (Yoon et al., 2014). Specific markers of MSCs, i.e., CD9, CD63, CD81, CD109, CD151, CD248, and CD276 as well as surface receptors (PDGF-RB, EGF-R, and PLAUR) involve in tissue recruitment and signaling molecules (RRAS/NRAS, Wnt5B, MAPK1, GNA13/GNG12, RHO, CDC42, and VAV2) controlling self-renewal and cell differentiation of human BM- MSCs have been reported (Kim et al., 2012). Consistent with the previous finding that EVs proteome include the proteins associated with EVs biogenesis and trafficking. Proteins implicated in intracellular transport and fusion, i.e., RAB are also very often present in EVs (Kowal et al., 2014, review). RAB proteins are accompanied with granule secretion, Golgi apparatus transport, tight junctions formation and ligand sequestration at the plasma membrane. These proteins, i.e., RAB1A, RAB2A, RAB5A/B/C, RAB7A, and RAB8A regulate docking and fusion of MVs with the recipient cell as well as proper targeting of them to various cellular compartments. Functional properties are also represented by proteins engaged in BM-MSCs-MVs cell adhesion (FN1, E2R, IQGAP1, CD47, LGALs1/LGALS3, and integrins), migration, and morphogenesis (Kim et al., 2012).

Lai et al. (2012) detected many important groups of peptides, i.e., ACTA, Alix, ANX, HSP, TUB, and TWHA that are secreted in the regular manner and be present in MVs derived from human ESC-MSCs. Some of these molecules were also characterized by Thery et al. (2009) in MVs derived from immune cells. Among them tetraspanins, clathrin, annexins, GAPDh, PK, EEF1A1, MFGES, MHC class I, cofilin1, ezrin, radixin, moesin, actin, and tubulin found in at least 50% of all examined exosomes (Thery et al., 2009). Nowadays, there are two public databases: EVpedia and ExoCarta containing data of EVs components of different cell types from several studies (Mathivanan et al., 2012; Choi et al., 2013). Similar to exosomes from other sources, protein components in MSC-derived exosomes do not remain constant due to heterogeneity of MSCs. Moreover, variations in the cell preparation have an influence on secretome profile of MSC derived from different sources (Lavoie and Rosu-Myles, 2013). The differences in protein content have been also detected in various batches of MSC-EVs (Yu et al., 2014). The proteomic analysis of EVs derived from MSCs isolated from different sources reveals distinguishing features from EVs derived from other types of cells (Skalnikova et al., 2011) and among microvesicles originated from MSCs of varying sources. Only a few studies have identified the whole proteome contained in MSC-derived EVs (Kim et al., 2012; Lai et al., 2012). However, the functional differences between EVs originated from distinct MSC sources clearly indicate the existence of difference in their composition. In both, *in vitro* dorsal root ganglia neurons and cortical neuron cultures cells react differentially to treatment with bone narrow (BM), umbilical cord blood (UCB), chorion (Cho-SC) and human menstrual fluid (MenSC) MSCs derived-exosomes. From all mentioned vesicles only MenSC -exosomes are able to enhance neurit outgrowth in cortical neuron cultures, while Cho-SC-exosomes cause even decrease of total neuron branch number. Moreover BM- and MenSC-derived exosomes increased the rate of neuritic growth in dorsal root ganglia neurons culture in comparison to control cells (Lopez-Verrilli et al., 2016). Similar observations were made in case of glioblastoma research. Among microvesicles (MVs) acquired from BM, UCB, and adipose tissue (AT) MSC only BM- and UCB-derived MVs decreased proliferation rate of glioblastoma cells line, whereas AT-MSC MVs had opposite effect. Induction of neoplasm cells apoptosis was observed after treatment with microvesicles from BM and UCB-MSC with no result in case of AT-MSC MVs (Del Fattore et al., 2015b). Furthermore these functional differences have been demonstrated even between vesicles from the same source but belonging to other sub-populations. Exosome-enriched fraction derived from BM-MSCs enhanced neurite outgrowth whereas the microvesicle-enriched fraction showed inhibitory effect (Lopez-Verrilli et al., 2016). The realization of more comparative studies between EVs derived from MSC from different sources is required. Based on these data it appears that MSC-EVs hold many characteristics of the MSCs themselves. Interestingly metalloproteinase inhibitors TIMP-1 and TIMP-2 were expressed only in human BM-MSC-EVs but not in parental cells (Vallabhaneni et al., 2015). In literature we can find a few examples of proteins which were present in microvesicles although they were not detected in cells of their origin. Authors of these articles associate this phenomenon with existence of very precise proteins sorting system during microvesicles biogenesis or limitation of protein identification techniques (**Table 2**) which very often suffer from high detection threshold or necessity of normalization of obtained results to the total protein level.

**Table 2:** Examples of articles with identification of differences between proteins composition in cells and vesicles originated from them.

Protein	EVs type	Differentiating feature			Authors’ explanation, comment	Reference
				
		Type	Cells of origin	Derived EVs		
NEP	ADSC-derived exosomes	Enzymatic activity	∼40% of total activity	∼90% of total activity	Unknown reason, it is likely that NEP was enriched in exosomes during their biogenesis	Katsuda et al., 2013
NEP	BM-MSC-derived exosomes	Enzymatic activity	**weak/undetectable**	70% of total activity		
CD63	ADSC-derived exosomes	Concentration	Higher	Weaker	CD63, CD81 are the well-established exosomal markers; Cyt-c and actin are typical cellular proteins	


CD81			**Weak/undetectable**	Present		
Cyt-c			Present	Weak/undetectable		
Actin			Present	Weak/undetectable		
LAMP2	BM-MSC vesicular fraction 40–150 nm	Concentration	Weaker	Higher	Results indicate the existence of regulated packaging mechanism of EVs with proteins that may not be highly expressed in cells; This research were done using western blot technique in which result is normalized to the same amount of total protein, the data seen is function of ratio of specific protein to total number of proteins	Vallabhaneni et al., 2015
CD90			Weaker	Higher		
PDGFR-β			Higher	Weaker		
CD9			**Weak/undetectable**	Present		
CD81			**Weak/undetectable**	Present		
TIMP-1			**Weak/undetectable**	Present		
TIMP-2			**Weak/undetectable**	Present		
Galectin-1	BM-MSC microvesicles	Concentration	Higher	Weaker	Typical microvesicles characteristic	Kim et al., 2012
HSP90			Higher	Weaker		
CD63			Higher	Weaker		
β-actin			Higher	Weaker		
CD63	MenSC microvesicles	Concentration	Weaker	Higher	The non-exosomal protein Rab5 was not detected	Lopez-Verrilli et al., 2016
TSG101			Weaker	Higher		
Hsp70			Weaker	Higher		
Hsp90			Weaker	Higher		
Rab5			Present	Weak/undetectable		


## Lipidomic Analysis of EVs

Except proteins EVs contain bioactive lipids. As for proteins, the lipid composition in EVs is distinct from that of the cell origin. Internal membranes of EVs isolated from different cell types are enriched in lysobisphosphatidic acids that modulate budding process and lipids associated with lipid rafts such as cholesterol, ceramide, sphingolipids, and glycerophospholipids with saturated fatty-acyl chains (Urbanelli et al., 2015). Sphingomyelin and cholesterol allow the tight packing of lipid bilayers and increase rigidity and stability of EVs derived from different cells, prevent their recognition by blood components and uptake, facilitate the fusion of EVs (Yoon et al., 2014). EVs also contain many lipid mediators such as prostaglandins and enzymes involved in their synthesis from membrane phospholipids. Subra et al have shown the presence of a set of phospholipases (A2, C, and D) in EVs isolated from RBL-2H3 cells. Also a large panel of free fatty acids including arachidonic acid were detected in EVs from mast cells (Subra et al., 2010).

## Profiling RNA Content in EVs

The RNA cargo has been well established as a component of EVs isolated from different cell types (Ratajczak et al., 2006). Various RNA species have been detected within EVs. The micro RNA (miRNA) is most abundant RNA species in human plasma and makeup over 76% of all mappable reads (Huang et al., 2013). Detailed analysis has shown that actually not mature miRNA but precursor miRNA (pre-miRNA) is mostly present in exosomes isolated from ESC-MSCs (Chen et al., 2013). There were found profound discrepancies between the exosomal and cellular content of miRNA suggesting an active process of sorting and packaging of miRNA into exosomes (Zhang J. et al., 2015). MSC-derived exosomes also contain significant amount of transfer RNA (tRNA), with striking differences in content between cells of AT or bone marrow origin, while no difference in miRNA content between these two cell sources has been found (Baglio et al., 2015). This may account for the differences observed between them (Muhammad et al., 2015). The translatable and fragmented mRNA is also present in EVs from different biological fluids (Rani et al., 2011). The RNA content varies depending of the exosome origin, for instance the fragments of ribosomal RNA (rRNA) was the most abundant RNA species found in breast cancer-derived EVs (Jenjaroenpun et al., 2013). The piwi-interacting RNA (piRNA) has been also recently detected in exosomes isolated from human saliva (Ogawa et al., 2013). It was recently found that EVs may not only cargo RNA, but also process it. Such process was observed in cancer-derived EVs and processed RNA was toxic for primary human cells (Chakrabortty et al., 2015). The high interest in RNA cargo of exosomes takes advantage of new methods of RNA isolation, which may bring more detailed characterization in near future (Enderle et al., 2015).

## Mechanism of Cellular Uptake of EVs

Extracellular vesicles released from parental cells may be broken down, thus releasing their content into extracellular space or neighbor cells may internalize them. The pathways through which EVs enter target cells impact EV-mediated biomolecule delivery. Several types of interactions between EVs and target cells have been demonstrated. Interaction may be direct, resulting in MVs fusion or endocytosis, i.e., observed between mouse dendritic cells (Mathivanan et al., 2010; Montecalvo et al., 2012) or indirect, by binding to surface receptors as visualized between tumor and immune cells (Clayton and Mason, 2009; Pan et al., 2014). EVs uptake is initiated by specific receptor-ligand interaction. The receptor-ligand binding is determined by several molecules such as integrins, tetraspanins and galectins and other adhesion molecules present on EVs isolated from different cell types and cell surface. (Rana et al., 2011; Raposo and Stoorvogel, 2013). The pattern of their expression is consistent with that of the cell origin. The correct orientation of these receptors enables their capability of encountering multiple ligands after their secretion from the cell (Morel et al., 2004). The presence of β1 and β2 integrins on exosomes of various cellular sources was shown. Clayton et al demonstrated that exosome integrins derived from B cells are capable of interactions with surrounding ECM and adhere to the ECM components such as collagen and fibronectin (Clayton et al., 2004). These adhesive interactions may limit diffusion of exosomes from the site of secretion. In inflammation or tissue injury, disruption of ECM leads to release ECM-bound exosomes liberating them to interact with resident or inflammatory cells expressing up-regulated adhesion molecules, i.e., ICAM-1, LFA-1, TIM-1, or TIM-4 (Thery et al., 2009). EVs may also potentiate ECM digestion through their inclusion or activation of matrix metalloproteinases (MMPs) such as MMP-2 and MMP-9 (Candela et al., 2010). Some studies revealed that EVs derived from platelet contained cytokine receptors (TNF-RI, and TNR-RII), platelet endothelium receptors (CD41, CD61, and CD62) and special ligands (CD40L, and PF-4) which could be transported into the target cells and enable platelet adhesion (Baj-Krzyworzeka et al., 2002). Furthermore, platelet derived EVs are able to interact with monocytes and endothelial cells but not with neutrophils. (Lösche et al., 2004) whereas EVs derived from neutrophils interfere with endothelial cells, monocytes and dendritic cells (DCs; Gasser et al., 2003; Eken et al., 2008). Once attached onto plasma membrane EVs moved in a slow drifted mode, then the motion mode changed to a rapid directed mode, indicating that EVs internalization occurred (Tian et al., 2013). After cellular uptake EVs are segregated within endosomes and fuse with lysosomes for degradation or with endosome membranes thus releasing their cargo into the cytoplasm (Turturici et al., 2014). Other studies revealed the evidence for accumulation of EVs in phagocytic or endocytic compartments and suggest that EVs uptake depends on the actin cytoskeleton, dynamin-2 and phosphatidylinositol 3 kinase activity (Raposo and Stoorvogel, 2013).

## Biological Activities of EVs

A very large collection of evidence shows that EVs are important regulators of many biological functions such as tissue homeostasis and immune response. EVs may influence the behavior of target cells by several different mechanisms. First they may act as signaling complexes. Indeed, EVs express several surface molecules, i.e., ICAM-1 interacting with the specific receptors LFA-1 present on T cells or δ-like 4 ligand bound to Notch receptors expressed by endothelial cells and neuronal cells thus activating these cells (Nolte-‘t Hoen et al., 2009; Biancone et al., 2012). EVs play an important role in signaling and morphogenesis during development. It was demonstrated that certain morphogenes, i.e., sonic hedgehog or retinoic acid associated with the epithelial cell membrane were released via vesicles in response to FGF signaling (Greco et al., 2001).

Extracellular vesicles may also transfer receptors, proteins, or bioactive lipids between cells after fusion with the target cell membrane. For example, the chemokine receptor CXCR4 or CCR5 could be transferred from lymphocytes to nonlymphoid cells (Rozmyslowicz et al., 2003). EVs-mediated transfer has been also described for adhesion molecules between platelets and hematopoietic cells (Baj-Krzyworzeka et al., 2002). Another biological activity of EVs is connected with delivering proteins to target cells. EVs may modulate the function of target cells by transferring intracellular proteins. By convey of pro-angiogenic factors, i.e., platelet-derived growth factor (PDGF), vascular endothelial growth factor (VEGF), basic fibroblast growth factor (BFGF) or leptin. EVs derived from CB-MSCs, BM-MSCs or shed from tumor cells can activate angiogenesis (Taraboletti et al., 2006; Zhang et al., 2012; Bian et al., 2014; Chen et al., 2014). It has been shown that EVs derived from activated monocytes are able to regulate apoptosis in target cells by transferring caspase-1 (Sarkar et al., 2009). Similarly, EV-related lipids induce several biological responses. The glycosphingolipids present in intracerebrally administered exosomes bind to beta-amyloid and clear the brain and decrease pathology in mouse model of Alzheimer disease (Yuyama et al., 2014). However, beta-amyloid induces the incorporation of C18 ceramides to EVs produced in astrocytes, which in turn have pro-apoptotic properties (Wang et al., 2012). In turn, the gangliosides GM1 and GM3 present in exosomes isolated from neuroblastoma cells facilitate the aggregation of alpha-synuclein, a protein involved in development of Parkinson disease (Grey et al., 2015). The role of lipids was not specifically investigated in MSC-derived EVs.

The miRNA mediates many biological effects through inhibition of specific mRNA (Huang et al., 2015). For example, miR-16 was capable to downregulate VEGF in breast cancer cells (Lee et al., 2013). Human ESC-MSC-derived exosomes are especially abundant with let-7 family of miRNA, which through HNF4A suppression contribute to maintenance of renewal of recipient stem cells (Koh et al., 2010). It was shown that mRNA shuttled between cells is functional and BM-MSC-derived exosomes transfer IGF-1R mRNA based on which protein is produced (Tomasoni et al., 2013). However, the most of mRNA present in exosomes is highly fragmented and cannot serve as a template for protein production, but it was found a very specific pattern of mRNA fragmentation within exosomes with enrichment in the 3′-intranslated regions. Since these regions are rich in miRNA binding sites, exosomal mRNA can compete with intracellular miRNA and disinhibit the mRNA translation (Batagov and Kurochkin, 2013). No specific biological activity of piwi-interacting RNA, tRNA, and rRNA present in exosomes has been reported.

## General Considerations on the Role of EVs in the Function of Immune System

Extracellular vesicles are involved both in promoting and in inhibiting the immune response, depending on their cell of origin and on the signals present in the microenvironment.

Macrophages infected by various pathogens (Mycobacterium and Toxoplasma) release EVs containing pathogen-derived pro-inflammatory molecular determinants inducing the secretion of pro-inflammatory cytokines by recipient macrophages (Bhatnagar and Schorey, 2007). Mycoplasma infection results in the release of EVs inducing polyclonal activation of B and T cells (Quah and O’Neill, 2007). EVs isolated from body fluids could exacerbate autoimmune diseases. In Rheumatoid Arthritis patients, fibroblasts isolated from synovial fluid secrete EVs expressing TNFα, which promotes survival of T lymphocytes (Zhang et al., 2006). EVs isolated from bronchoalveolar fluid of patients with sarcoidosis stimulates the secretion of pro-inflammatory cytokines by epithelial cells (Qazi et al., 2009).

Extracellular vesicles secreted by DCs can either promote or inhibit immune response depending on the degree of maturation of their parent cells. EVs produced by mature DCs carry both antigenic material and MHC-peptide complexes required for the initiation of immune responses by APCs. In addition, secreted vesicles also express co-stimulatory molecules. More efficient T cell activation was obtained with exosomes purified from mature, rather than immature, DCs, suggesting that costimulatory molecules present in EVs play indeed a role in the immune response (Admyre et al., 2003). Such EVs are not only capable of presenting antigens directly to T cells but are also able to transfer both the MHC II molecule and the antigen to naïve DCs thus amplifying the immune response (Thery et al., 2002). EVs from mature DCs primed with male antigen peptide enhance male skin graft rejection by female mice (Segura et al., 2005). *In vitro* priming of DCs with specific antigens results in the production of EVs which can induce *in vivo* humoral responses against the same antigens (Clayton et al., 2001; Aline et al., 2004; Qazi et al., 2009), stimulating both T and B cells, leading to both memory Th1 and immunoglobulin responses (Qazi et al., 2010). A promoting effect on NK activity was observed in clinical trials of cancer patients treated with EVs from their own DCs primed *in vitro* with their cancer cells (Escudier et al., 2005; Viaud et al., 2009).

Tumor-derived EVs can also play opposite roles in immune response, depending on yet poorly identified mechanisms. Cancer EVs can stimulate the immune response by transferring tumor antigens to DCs (Wolfers et al., 2001), leading to Ag-specific T cell activation, in particular of CD8 cytotoxic T lymphocytes (CTL) clones (Hsu et al., 2003; Utsugi-Kobukai et al., 2003; Chaput et al., 2004; Escudier et al., 2005). Although tumor-derived EVs can prime DCs to stimulate the immune response, they can also behave as immunosuppressive (Poutsiaka et al., 1985; Clayton et al., 2007) favoring cancer escape from immune surveillance. Tumor-derived EVs can induce T cell apoptosis via FasL (Andreola et al., 2002; Huber et al., 2005) and galectin-9 (Klibi et al., 2009), inhibit IL-2-induced T cell proliferation (Thery et al., 2002), promote Tregs (Szajnik et al., 2010), reduce CD8+ T cells proliferation (Wieckowski et al., 2009) and decrease NK cell cytotoxicity (Ashiru et al., 2010), as well as induce myeloid supressor cells (Cocucci et al., 2007).

Vesicles secreted by immune cells can also display immunosuppressive properties. As mentioned above, EVs secreted by immature DCs can induce tolerogenic, rather than effector immune responses (Peche et al., 2003). It was shown that such EVs promote graft survival (Peche et al., 2003) and reduce inflammation in animal models of arthritis (Kim et al., 2005), of inflammatory-bowel disease (Yang et al., 2010) and of septic shock (Miksa et al., 2006, 2009). Activated T cells secrete exosomes bearing FasL, which induce apoptosis of neighboring T cells, suggesting their participation in the regulation of immune response by a negative feedback mechanism (Monleon et al., 2001). Interestingly, placenta secretes EVs which seem to contribute to fetomaternal tolerance (Taylor et al., 2006). Exosomes in plasma of pregnant women bear FasL and reduce CD3ζ expression by T cells (Taylor et al., 2006) as well as NKG2D ligands, reducing the cytotoxicity of NK and CD8+ T cells (Hedlund et al., 2009).

## Immunomodulatory Role of MSC-Derived EVs

Recent studies indicate that the immune modulatory activity of MSCs can be at least partially mediated by their ability to release EVs. The inhibitory effects of MSCs on B-cell proliferation and differentiation in a CpG-stimulated peripheral blood mononuclear cell co-culture system could be fully reproduced by EVs isolated from MSC culture supernatants in a dose-dependent fashion (Budoni et al., 2013). A dose-dependent inhibitory activity of MSC-EVs was also observed for IgM, IgG, and IgA production. Moreover, in the same coculture system 7-AA-negative and Annexin-positive MSC-EVs isolated from mesenchymal stromal cells were internalized in a subset of CD86/CD19 positive cells corresponding to activated B lymphocytes. The effect of EVs on T cells was investigated by Mokarizadeh et al. (2012) in a rodent model. These authors showed that EVs isolated from murine BM-SCs inhibited the proliferation of both syngenic and allogenic T lymphocytes. Additionally, they demonstrated that these microparticles were able to induce apoptosis in activated T cells. Interestingly, this inhibition was associated with an increased proportion of regulatory T CD4+-CD25+-FoxP3+ cells. Moreover, an increased secretion of IL-10 and TGFβ1 by cultured splenic cells added with MSC-EVs was observed. These results suggest that MSC-EVs can induce tolerogenic signaling. Similar results were observed in human PBMC cultures treated with human T cell activator CD3/CD28 beads (Del Fattore et al., 2015a). Stimulation increased the number of proliferating CD3+ cells as well as of T regulatory cells (Treg). Co-culture with MSCs inhibited the proliferation of CD3+ cells, with no significant changes in apoptosis. Addition of MSC-EVs to PBMCs did not affect proliferation of CD3+ cells, but induced the apoptosis of CD3+ cells and of the CD4+ sub-population and increased the proliferation and the apoptosis of Treg. Moreover, MSC-EV treatment increased the Treg/Teff ratio and the immunosuppressive cytokine IL-10 concentration in culture medium. The activity of indoleamine 2,3-dioxygenase (IDO), an established mediator of MSC immunosuppressive effects, was increased in supernatants of PBMCs co-cultured with MSCs, but was not affected by the presence of MSC-EVs. Interestingly, MSC-EVs express Galectin-1 and PD-L1 (Garin et al., 2007; Kilpinen et al., 2013) two molecules also expressed on MSC surface (Kadri et al., 2005; Pedemonte et al., 2007). Galectin-1, an endogenous leptin, has been shown to induce apoptosis of activated T cells (Rabinovich et al., 2000) and to promote the generation of Treg (Blois et al., 2007) PD-L1, a negative costimulatory molecule for PD-1, also promotes Treg proliferation and function (Freeman et al., 2000; Chen et al., 2003). Moreover, MSC-EVs express TGF-alfa, a well-known inducer of Treg (Chen et al., 2003).

*In vitro* results are also supported by *in vivo* observations in an animal model of inflammatory bowel disease (Del Fattore et al., 2014) induced by dextran sulfate sodium (DSS). Mice injected daily with MSC-EVs showed less weight loss, improved disease activity index and a less severe reduction in colon length when compared to DSS/vehicle-treated controls. qRT-PCR analysis performed on RNA extracted from colon tissue revealed a strong inhibition of the induction of inflammatory cytokines with respect to untreated animals. Collectively, these data suggest that EVs isolated from MSCs could reproduce the immunomodulatory effect of MSCs. Indeed, MSC-EVs are attracting increasing interest since they might represent a more convenient therapeutic tool with respect to their cells of origin. Interestingly, a case of successful treatment with MSC-EVs in a patient with steroid-resistant GVHD was recently reported (Kordelas et al., 2014). However, additional work both *in vitro* and *in vivo* is needed in order to better understand both the potency and the mechanisms of action of this novel potential immunosuppressive tool.

## EV-Mediated Immunomodulation in Neurological Disorders

The use of EVs for immunomodulation of neurological disorders is still in its infancy, however, several attempts have been devised. Genetically modified DCs to equip EVs with TGF-β1 inhibited the progression of murine experimental autoimmune encephalomyelitis (EAE; Yu et al., 2013). Immature DC-derived exosomes ameliorated the progression of experimental autoimmune myasthenia gravis (Bu et al., 2015). Exosomes derived from atorvastatin-modified DCs ameliorated experimental autoimmune myasthenia gravis by up-regulating the levels of IDO and of Tregs and shifting Th1/Th17 to Th2 cytokines (Li X.L. et al., 2013, 2016). The mesenchymal stromal cell-derived EVs rescued traumatic brain injury (TBI)-induced cognitive impairment in part through reducing of neuroinflammation (Zhang Y. et al., 2015; Kim et al., 2016).

## EVs in the Brain Neural-Glial Networks

In the nervous system EVs are released by many cells including cortical and hippocampal neurons, glial cells, astrocytes, and oligodendrocytes and that EVs have significant impact on communication within the CNS. EVs present in extracellular and cerebrospinal fluids transfer protein, lipid and nucleic acid cargo from one cell to another modifying the target cell phenotype and function (Agnati et al., 2010). Several lines of evidence reveal that EVs relay complex messages other than (or even superior to) those based on direct cell-to-cell contacts or secreted soluble factors.

In neurons, EVs shed at the synapses are implicated in *trans* synaptic communication. They can be retaken by other neurons suggesting a novel way for inter neuronal communication. The first evidence of EVs release from neural cells was demonstrated *in vitro* using primary culture of embryonic cortical neurons isolated from rats and mice (Fauré et al., 2006). Additional studies reported secretion of EVs from fully differentiating cortical cells, which contained glutamatergic and GABAergic neurons in long-term culture (Lachenal et al., 2011). In mammalian cortical neurons EVs are predominantly distributed within somatodendritic compartment, where they are 50 times more represented than in axons (von Bartheld and Altick, 2011). EVs deriving from this compartment may exert alternating functions at the level of synapses. Indeed in neurons, EVs are present in both pre- and postsynaptic components. Studies on trafficking of synaptic AMPA type receptors, which represent the main mediators of fast synaptic transmission among glutamate receptors of the CNS showed that neuronal EVs act as stores for synaptic receptors (Kennedy and Ehlers, 2006). As neuronal EVs carry AMPA receptor subunits they may play a role in synaptic plasticity by regulating the AMPA receptors for glutamate transmission (Chivet et al., 2013). Moreover, EVs can transport functionally competent GPCRs adding a further level of plasticity forming the receptors that acquire the ability to respond to its neurotransmitter ligand (Guescini et al., 2012). It was shown that increasing cytosolic calcium, incubation with GABA receptor antagonists or neuron depolarization increased EVs secretion (Lachenal et al., 2011; Chivet et al., 2012; Pegtel et al., 2014).

In addition to neurons, other cells in the CNS release higher amount of EVs. Astrocyte-derived EVs are heterogeneous and their composition depending on the environment. A large number of transfer compounds such as mitochondria, mitochondrial DNA, ATP, glutamate transporters, Hsp/Hsc70 and synapsin I involved in neuroprotection, factors modulating angiogenesis, i.e., FGF2, VEGF, PEDF, and endostatin, as well as MMPs mediated ECM proteolysis have been identified in astrocytic EVs (Frühbeis et al., 2013; Agnati and Fuxe, 2014; Pegtel et al., 2014). The target cells are both astrocytes and neurons, dependent on the cargo in EVs they may produce, being involved in neuronal growth and survival, synaptic transmission regulation or degeneration. Astrocytic EVs can contain excitatory amino acid transporters that may have special function in volume transmission by scavenging glutamate in the extracellular fluids, reducing excitation, and neurodegeneration (Agnati and Fuxe, 2014).

Microglial provide the first line of defense during infection and brain injury. Upon stimulation reactive microglia release EVs that transmit inflammatory signals to recipient microglia which then upregulate the expression of genes enhancing inflammation, i.e., IL-1β, IL-6, iNOS, cyclooxygenase etc. (Verderio et al., 2012; Prada et al., 2013). Thus microglial EVs spread inflammatory reactions throughout the brain. It is of interest that microglia-derived EVs can interact with neurons and enhance excitatory transmission modulating synaptic activity (Antonucci et al., 2012). In addition microglia release EVs with protein content previously reported in B cell- and DC-derived EVs. Although MHC class II antigens are visualized in microglial EVs, their relevance for antigen presentation exhibited by microglia themselves is still open (Potolicchio et al., 2005).

Oligodendrocytes produce the myelin sheath around axons thus facilitating impulse conduction. Recent studies suggest that these trophic functions may depend on the transfer of EVs from oligodendrocytes to neurons. Indeed, the oligodendrocyte EVs contain myelin proteins such as PLP, CNP, MAG, and MOG (Krämer-Albers et al., 2007; Frühbeis et al., 2013). The secretion of EVs from oligodendrocytes is regulating by neurotransmitter signaling. Axonally released glutamate activates EVs release from oligodendrocytes mediated mainly by NMDA receptors. In addition, oligodendrocyte-derived EVs have been suggested to negatively regulate myelin synthesis in an autocrine manner (Bakhti et al., 2011). However, Fruhbeis and colleges did not observe PLP-positive EVs in myelinating fibers *in situ* and postulated that EVs derived from oligodendrocytes are released into the periaxonal space and thus involve in axon-glia interaction (Frühbeis et al., 2013). Moreover, oligodendrocyte EVs improve the metabolic activity of cultured neurons under cell stress by delivery of supportive biomolecules. This is the evidence that EVs released from oligodendrocytes participate in bidirectional neuron-glial integrity.

**FIGURE 2:**
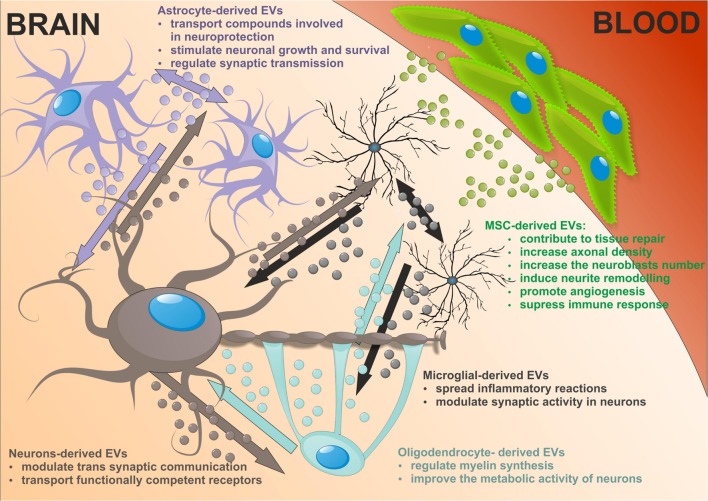
**The neuronal-glial networks signaling using EVs with potential impact of mesenchymal stem cells (MSC)-derived EVs treatment on nervous tissue regeneration**.

Extracellular vesicles released by neural cells into brain parenchyma could be potentially endocytosed by nearby cells. Then EVs cargos are released into the cytosol of receiving cell or re-express at the cell surface. Astrocytes, which enwrap a number of glutamatergic synapses could capture EVs released at synapses. Back-fusion EVs has been demonstrated to occur in CNS and could concern their cells of origin. On the other hand EVs released from particular neural cells can be engulfed by other type of cells in CNS. EVs secreted by neurons may be transferred between spines of the same neuron or across synapses to end up in afferent neurons. Microglial EVs are able to be internalized by the same cell or by the neighboring microglia in macro-pinocytic fashion (Chivet et al., 2012). Oligodendrocyte-derived EVs are usually taken by neurons. This uptake seems to be selective since astrocytes and oligodendrocytes internalize oligodendroglial EVs to a minor extent. There is an evidence that oligodendrocytes also interact with and respond to microglia via releasing EVs that are taken by recipient cells (Peferoen et al., 2013; **Figure 2**).

## EV-Based Strategies for Diagnosis of CNS Diseases

Extracellular vesicles are increasingly gaining attention in diagnostic tools, being used as potential biomarkers for the detection of early pathological conditions before the onset of clinical symptoms of the disease. The choice of potential sources for EVs includes blood, plasma and cerebrospinal fluids. Relative stability of EVs in body fluids and their ability to pass the blood–brain barrier have suggested to exploit them very easy with minimally invasive procedure (so called “liquid biopsy”). Finding and testing such biomarkers in parallel with other diagnostic tools might be very important in CNS disorders to understand complex neurological conditions.

The molecular content of EVs namely proteins, nucleic acids, and lipids reflects the origin and the pathophysiological status of the releasing cells. Several studies have demonstrated that EVs isolated from body fluids of neurological patients comprise molecules implicated in neurodegenerative diseases, metabolic, infectious diseases, or cancer. The concentration of EVs increases upon inflammation accompanied different neurological diseases and it is closely related with disease course (Lee et al., 1993; Verderio et al., 2012).

Certain EV proteins provided a tool to distinguish a disease-related condition from a healthy state. For example, amyloid precursor protein fragments (APP) or tau phosphorylated at Thr181 are established biomarkers for Alzheimer’s disease, and phosphorylated tau or α-synuclein are relevant for Parkinson’s disease (Saman et al., 2012; Yang et al., 2015). The presence of scrapie form of the prion protein (PrPSC) in isolated EVs is usually the evidence of Creutzfeld-Jacob disease (Coleman et al., 2012; Coleman and Hill, 2015). Many recent reviews addressed different EVs-derived proteins as of brain tumors diagnosis (Pegtel et al., 2014; Kawikova and Askenase, 2015; Paschon et al., 2015). These proteins are usually present on EV surface and could be general cancer markers, cancer-type markers or tissue-type markers. Plasmatic levels of cancer-derived EVs were reported to be related to the tumor size, the metastatic behavior of tumor (calveolin-1) or their impact on angiogenesis, pro-survival, apoptosis, immunomodulation, or drug resistance (D’Asti et al., 2012; Redzic et al., 2014). In particular, the persistence of tumor-specific exosomes in body fluids such as blood after resection of tumor on the primary site can indicate the existence of metastases, which could be located in various organs including brain and may spur further diagnostics and treatment. The examination of EVs content in blood samples from control and glioblastoma patients was performed by Shao et al. (2012). They created a very innovative system in which isolated EVs were labeled with magnetic nanoparticles reacting with specific proteins and then identified by the miniaturized nuclear magnetic resonance system. This strategy allowed to identify with high detection sensitivity glioblastoma-secreted exosomes and to distinguish them from EVs originating from healthy individuals.

The disease-specific proteins secreted in EVs or incorporated into their membrane were found in *in vitro* research in response to cells infection by viruses spreading on CNS like human immunodeficiency virus 1 (etiological cause of Acquired Immune Deficiency Syndrome), human T-cell leukemia virus-1 (evoking tropical spastic paresis), herpes simplex virus-1 (inducing herpes viral encephalitis; Sampey et al., 2014).

Several studies have revealed genetic alterations in RNA in EVs derived from neurological disorders in comparison with healthy individuals (Rao et al., 2013; Mundalil Vasu et al., 2014). Profiling RNA expression patterns could facilitate presymptomatic disease detection. Recent reports point out EVs nucleic acids as the biomarkers corresponding to brain injury, neurodegenerative diseases, neuroinflammation, or brain tumors. Exosome Diagnostic (Cambridge, MA, USA) filed a patent reporting a technique to detect neurodegenerative diseases and brain injury based on the measure of RNA-s (mRNA, miRNA, siRNA, or shRNA) associated to CSF-derived EVs (Skog and Russo, 2015). In the reported examples, biomarkers associated with different neurodegenerative diseases were nucleic acids corresponding to APP, Aβ42, BACE1, and tau protein (Urbanelli et al., 2015). The study of Skog et al. (2008) revealed the presence of oncogenic acids in EVs derived from brain tumors into CNF. Molecular analysis of oncosomes shed from brain tumor cells indicates the presence of mRNA coding mutated genes, non-coding RNA (multiple miRNAs), transcripts for different oncoproteins or oncogenic DNA sequences. Amongst the described examples the point mutation in gene coding epidermal growth factor receptor (EGFRvIII) was found in EVs isolated from glioblastoma patients (Weller et al., 2014). Similarly, mRNA for mutated form of IDH1/2 gene and mRNA for abnormal C-myc gene were observed in EVs circulating in blood of glioma and medulloblastoma patients, respectively (Balaj et al., 2011; Chen et al., 2013). Recently, EVs from glioblastoma and astrocytoma have been shown to carry mtDNA and dsDNA representing the whole genomic DNA, which can be used to identify mutations present in tumor cells (Thakur et al., 2014).

As stated above, EVs are involved not only in physiological processes but also in CNS diseases carrying specific pathologic cargo. Over the last decades, besides of using them as biomarkers of different diseases EVs have been proposed as therapeutic tools for neurological disorders.

## Non-Invasive Imaging of EVs

Mesenchymal stem cells have the ability to release several pro-survival trophic and immunomodulatory factors (Abboud et al., 1991; Aggarwal and Pittenger, 2005; Wilkins et al., 2009; Jitschin et al., 2013). Because of these beneficial properties, MSCs have been successfully used in experimental animals to treat several neurological disorders and to improve graft survival in the CNS (Srivastava et al., 2016). Human MSCs derived from BM or UCB were shown to have a strong capacity for exosome secretion in response to cellular injuries (Baglio et al., 2012; Li T. et al., 2013) and the pro-survival and immunomodulatory effects of these cells may be attributable to exosome release. The imaging of exosomes *in vivo* may contribute to an understanding of the regenerative potential of exosomes released from MSCs and would also represent a significant advancement in translational exosome science.

The ability to non-invasively track exosomes *in vivo* using different imaging modalities is still in its infancy. Because of their nanometre size, the traditional method of visualization of exosomes is scanning electron microscopy (SEM; Sharma et al., 2010; Sokolova et al., 2011). SEM allows particle size determination, and therefore helping to distinguish between exosomes and other vesicles. Fluorescence nanoparticle tracking analysis (NTA) has also been used to determine the exosome size on the basis of Brownian motion (Dragovic et al., 2011). Other methods include bright fluorescent labeling of cell-derived exosomes and high-resolution flow cytometry for quantitative and qualitative analysis (van der Vlist et al., 2012) and Tunable Resistive Pulse Sensing analysis, a high resolution technique that measures the change in electrical resistance in a pore as a particle passes through it (Coumans et al., 2014). However, these methods are not ideal for the visualization of *in vivo* localization and biodistribution of exosomes. In the last several years, the development of *in vivo* imaging techniques has significantly improved our ability to non-invasively track exosomes. With these techniques, we can now monitor the distribution of exosomes at the site of injury or elsewhere in the body.

Exosomes could be visualized by introducing a labeling agent, and then, imaging the labeling agent as a surrogate for the exosomes. Depending on the labeling agent, exosomes can be imaged by optical imaging, magnetic resonance imaging (MRI), or single-photon emission computed tomography (SPECT).

Lai et al. (2014) used an optical imaging approach to visualize exosomes. They labeled exosomes with *Gaussia luciferase* for non-invasive bioluminescence imaging (BLI). BLI is based on the emission of photons in reactions catalyzed by luciferase enzymes. Luciferases emit photons during the oxidation of a substrate, such as *D*-luciferin, in the presence of oxygen and ATP. BLI of immunodeficient, athymic nude mice systemically injected with exosomes showed a prominent bioluminescence signal in the spleen (Lai et al., 2014). In another study, Grange et al. (2014) used small-molecule near-infra red (NIR) fluorophores to label exosomes and track them for non-invasive visualization. They used two different exosome labeling protocols. In the first protocol, MSC-derived exosomes were directly labeled with Vybrant DiD during an ultracentrifugation procedure. In the second protocol, exosomes were indirectly labeled with fluorophores by incubating MSCs with a Vybrant DiD cell-labeling solution, and then, isolating exosomes from MSCs by ultracentrifugation. *In vitro* optical imaging showed a brighter fluorescence signal in exosomes directly labeled with DiD compared to exosomes obtained by MSCs that were previously labeled with DiD. *In vivo* optical imaging of mice with acute kidney injury, intravenously injected with directly or indirectly labeled exosomes, showed an accumulation of exosomes, especially in the kidneys (site of injury) of mice. Directly labeled exosomes showed a higher and brighter fluorescence compared to indirectly labeled exosomes. This study showed that both labeling methods were suitable for the *in vivo* detection of exosomes (Grange et al., 2014). However, one of the major limitations of optical imaging is light absorbance from hemoglobin and multi-layer anatomical barriers that limit the light emission.

Another method for exosome visualization is MRI. High spatial resolution and the ability to gather accurate anatomical information and image deep inside the tissue are some of the greatest advantages of MRI. Superparamagnetic iron oxide (SPIO) nanoparticles are MRI contrast agents that are conventionally used for cell-tracking. SPIOs are based on magnetite or maghemite cores embedded and stabilized with a hydrophilic shell. The SPIO core contains several million iron atoms. These particles create a large dipolar magnetic field (Hao et al., 2010) and signal spin-spin dephasing due to the local field inhomogeneity induced in water molecules near the particles (Rogers et al., 2006). This results in negative contrast on T2-weighted MRI. Hu et al. (2014) utilized this property to track exosomes by labeling them with SPIOs. They labeled mouse B16-F10 melanoma cell-derived exosomes with SPIOs by using electroporation. These SPIO-labeled exosomes were then injected into the footpad of C57BL/6 mice. MRI of these mice showed visually appreciable nodal enhancement and apparent enlargement 48 h after the injection (Hu et al., 2014). This study was a proof-of-principle study that demonstrated that exosomes can be tracked by MRI. One of the major disadvantages of this method is the possible release of iron particles from exosomes and their deposition in the tissue. Deposited iron particles could be scavenged by macrophages and may generate a false-positive signal on MRI.

**FIGURE 3:**
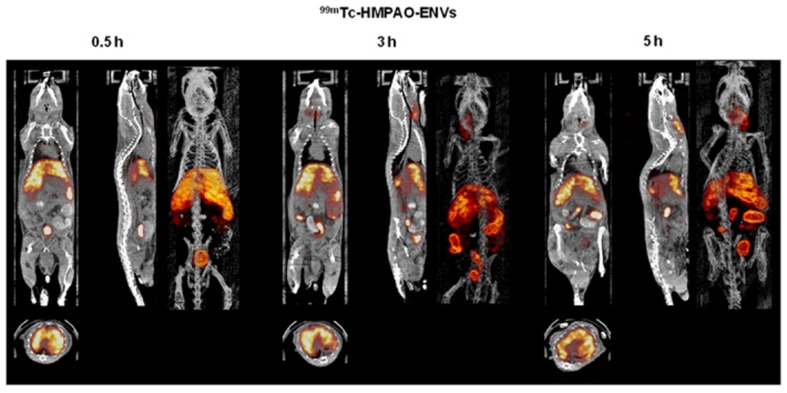
***In vivo* SPECT/CT images of ^99m^Tc-HMPAO-ENVs injected in mice.** After intravenous injection of ^99m^Tc-HMPAO-ENVs or ^99m^Tc-HMPAO, SPECT/CT images were acquired at 30 min, 3 h, and 5 h in BALB/c mice. The SPECT/CT imaging showed the significantly intense uptake of ^99m^Tc-HMPAO-ENVs in the liver and radioactivity in the salivary glands and intestine until 5 h. (Reproduced from Hwang et al., 2015.)

The nuclear imaging modality, SPECT, could also be used to image exosomes. Because of the superior tissue penetration capability of SPECT, and its more quantitative nature than optical imaging (Massoud and Gambhir, 2003), the use of SPECT for exosome imaging presents a better clinical potential. A study by Hwang et al. (2015) reported a simple method for radiolabelling of macrophage-derived, exosome-mimetic nanovesicles (ENVs) with ^99m^Tc-HMPAO (a clinically used tracer) under physiologic conditions, and monitored the *in vivo* distribution of ^99m^Tc-HMPAO-ENVs using SPECT/CT in living mice. SPECT/CT images exhibited a higher uptake of ENVs in the liver and no uptake in the brain (**Figure 3**; Hwang et al., 2015). Although this technique shows greater promise for investigating the *in vivo* behavior of exosomes and is more clinical applicable, the trade-off between half-life and long-term exposure to ionizing radiation and a possible transfer of radiometal to surrounding cells could be a major limitation of using ^99m^Tc-HMPAO for tracking.

## Therapeutic Potential of MSC-Derived EVs in CNS Disorders

While the use of MSCs in regenerative medicine raised high expectations in clinical settings, the use of MSC-derived vesicles released by these cells could have many advantages compared to a cell-based approach. Therapeutic potential of EVs seems to be more attractive because it reduces the risk associated with engraftment of cells, possible immune reactions against cells and emboli. Moreover, EVs have a unique ability to cross biological barriers as was shown in glioblastoma patients (Noerholm et al., 2012; Shao et al., 2012) what is very important for neurological disease therapy where the compounds that are administered systemically need to cross blood-brain barrier and blood-CSF barrier.

Extracellular vesicles may affect cell senescence, proliferation and cell survival reducing apoptosis resulting from brain ischemic injury. Besides, as EVs from MSCs were shown to modulate several signaling pathways, they could be used to treat neurodegenerative diseases or brain tumors. MSC-derived EVs have been reported to contribute to tissue repair using the experimental models of brain injury. Xin et al. (2013a) showed that systemic administration of EVs generated from bone marrow-derived MSCs significantly increased axonal density and synaptophysin-positive area in the ischemic cortex and striatum of middle carotic artery occlusion (MCAo) rats. BM-MSC-derived EVs treatment increased also the number of newly formed doublecortin positive cells (neuroblasts) and improved functional recovery of stroke rats compared with PBS-treated controls. The observation of Xin et al. (2013a) that BM-MSCs exposure to ischemic rat brain extract induced the expression of miRNA133b in MSC and the previous statement of Yu et al. (2011) that miRNA 133b was essential for the functional regeneration of motor neuron axons after spinal cord injury in zebrafish prompted the authors to check this effect *in vitro*. Treatment of primary rat cortical neurons with EVs-derived from ischemic brain extract-treated MSCs increased the total number of neurites and their length after 48 h (Xin et al., 2012). Further studies have demonstrated that BM-MSC-derived EVs transfer of miRNA133b into rats subjected to MCAo induced neurite remodeling, increased axonal plasticity and functional outcome of rats 14 days after stroke onset (Xin et al., 2013b). This effect has been selectively specific since GTPase RhoA, an miRNA 133b inhibitor usage did not change neurite morphology. This was the proof that EVs-mediated secretion of miRNA contributes to the protective effect of MSCs on stroke.

In addition to the beneficial effect on neurogenesis, EVs promote angiogenesis post stroke. Rats that received BM-MSC-EVs demonstrated a significant increase in the percentage of BrdU/vWF- positive cells in ischemic zone (Xin et al., 2013b). Cerebral endothelial cell proliferation contributed to neurovascular remodeling with the ischemic tissue. Therapeutic application of EVs obtained from human bone marrow derived mesenchymal stem cells (hBM-MSC) has been also shown in the experimental model of TBI in rats. Intravenous injection of secretome from hBM-MSC ameliorated TBI-induced rats by reducing neuronal cell loss in the injured cortex and promoting proangiogenic VEGF production resulting in functional outcome improvement (Chuang et al., 2012). Moreover, rats treated with EVs derived from BM-MSC of normal and cerebral ischemic rats had decreased infract volume in comparison to untreated animals. Similarly, the recovery of neurological functions after ischemic stroke was observed after application of condition medium of rat MSC derived from bone marrow, accompanied with the increase of neuronal progenitor cells surrounding lateral ventricle in stroke affected hemisphere (Tsai et al., 2014).

Recently, a new therapeutic possibility for using MSC-derived EVs against Alzheimer Disease was proposed by Katsuda et al. (2013). The authors have found that human AT-MSCs secrete nephrilysin (NEP), which is the most important β amyloid (Aβ)–degrading enzyme in the brain. The functional NEP – bound exosome derived from hAT-MSC decreased Aβ overexpressed by neuroblastoma cells in co-culture settings (Katsuda et al., 2013).

Apart from natural EVs, genetically engineered exosomes can be used as a delivery system for small molecule therapeutics for treating CNS diseases. EVs are proposed as ideal nucleic acid transporters. The study of Pusic et al. (2014) exploring the ability of DC-derived exosomes packed with miRNA showed a significant increase in myelination observed in hippocampal slice cultures subjected on oxidative stress. In another study, exogenous delivery of miR-124a by stereotactic injection of neuronal cell-derived EVs prevented pathological loss of GLT1 protein, an important glutamate transporter, selectively lost in amyotrophic lateral sclerosis, in SOD1 G93A mice the experimental model of ALS (Morel et al., 2013).

The neuro-oncologic application of EVs harnessed with miRNA has come from the observation that such modified BM-MSC-derived EVs were incorporated by tumors cells in co-culture system (Katakowski et al., 2010). Followed by *in vivo* studies, intra-tumor injection of endosomes-derived from miR-146 overexpressing BM-MSC significantly have been shown to reduce glioma xenograft growth in rat brain (Katakowski et al., 2013). Not only microRNA but also mRNA and siRNA have been overexpressed in donor cells and delivered by exosomes. Exosomes isolated from HEK-293T cells previously transfected with suicide mRNA triggered tumor cell apoptosis and tumor regression after their direct injection into schwannoma (nerve sheat tumor) in an orthotopic mouse model (Mizrak et al., 2013). EVs isolated from different cell types loaded with siRNA have also been shown to be successful cargo vehicles for siRNA delivery to the brain (El Andaloussi et al., 2013). The first *in vivo* example of how to exploit EVs carrying siRNA has come from Alvarez-Erriti studies. Systemic delivery of DC-derived EVs previously tackled with exogenous si-RNA induced knockdown of BACE1, a therapeutic target in AD in the mice brain (Alvarez-Erviti et al., 2011).

It is important to recall that EVs may supress the immune response. This strategy may provide a novel therapeutic approach for treating brain inflammatory-related diseases of CNS such as Parkinson’s disease, Alzheimer disease, multiple sclerosis, amyotrophic lateral sclerosis, meningitis, brain, spinal cord, and peripheral nerve injury and brain tumors. The anti-inflammatory effect of AT-MSC-derived EVs was shown to improve rat sciatic nerve regeneration after experimental transection (Raisi et al., 2014). Moreover, genetically modified EVs could deliver different immunosuppressant substances to modify immune reaction. The antioxidant curcumin-loaded exosomes isolated from murine macrophage cell line have been shown to decrease IL-6 and TNF levels *in vitro and in vivo* (Sun et al., 2010). In a separate study BV2 microglial cell line derived exosomes encapsulating signal transducer and activator of transcription 3 (STAT-3) inhibitor (JSI-124) were delivered to the brain and selectively taken up by microglial cells induced apoptosis (Zhuang et al., 2011).

A specific application of exosomes released by MSCs was patented by Beelen et al. (2014). The authors claimed that exosome preparations derived from neonatal and adult tissue-derived MSCs were effective for the therapy of inflammation pre- and post-natally acquired damages of the brain (Beelen et al., 2014). Another patent disclosed the preparation and use of exosomes isolated from neural stem cells induced from MSCs for the treatment of CNS diseases (Tian et al., 2013).

The use of EVs derived from MSCs allows also for avoiding risk related to the direct deposition of MSCs in the CNS such as formation of fibrotic masses (Grigoriadis et al., 2011; Snyder, 2011).

## Conclusion

The field of EVs is maturing with a fast progress in the untangling of structure and content of EVs. There are also advances in understanding of biological activities of EVs, in particular in cancer and in immunology-related diseases, but its role in CNS disorders also draws a lot of attention, recently. It translated to the attempts to use EVs as biomarkers of CNS disorders as well as therapeutic agents. MSs have been shown to be therapeutic in many neurological disorders despite the lack of homing to the CNS. Thus, there is growing appreciation of EVs as mediators of MSC-derived therapeutic effects. MSCs are easy to obtain and maintain, thus a lot of interest in now paid to replacement of MSCs by MSC-derived EVs as therapeutic agents. Altogether, the EVs shed a new light on physiology and pathology, as well as become an attractive source of therapeutic agents.

## Author Contributions

All authors declare the contribution in this paper. The authors were responsible for the following parts of the review: introduction (BL), types of EVs (SK and BL), proteomic analysis of EVs (SK and BL), profiling RNA content in EVs (MJ), mechanism of cellular uptake of EVs (SK and BL), biological activities of EVs (SK and BL), role of EVs in immune responses (MM), EVs in the brain neural-glial networks (AA and BL), EVs - based strategies for diagnosis of CNS diseases (AA and BL), non-invasive imaging of EVs (AS), therapeutic potential of MSC-derived EVs in CNS disorders (AA and BL), conclusion (BL).

## Conflict of Interest Statement

The authors declare that the research was conducted in the absence of any commercial or financial relationships that could be construed as a potential conflict of interest.
