# Supplementary material for: Extracellular Vesicles in Physiology, Pathology, and Therapy of the Immune and Central Nervous System, with Focus on Extracellular Vesicles Derived from Mesenchymal Stem Cells as Therapeutic Tools
Source: Front Cell Neurosci. 2016 May 2;10:109. doi: 10.3389/fncel.2016.00109 (PMC4852177; doi:10.3389/fncel.2016.00109)
Supplement: Supplementary file 1 [file Data_Sheet_1.PDF]

Supplementary data table 1. List of unique proteins identified in EVs by Lai et al. and Kim et al.

**Unique proteins list**											
**Lai et al., 2012**						**Kim et al., 2011**					
A2M	AEBP1	ALDOB	APP	ATRN	C1S	ABCC1	AIDA	AP2A2	ATIC	B2M	CAMK2D
ABI3BP	AFM	ALOX12P2	ARF5	ATXN1	C20orf114	ABHD14B	AK1	AP2B1	ATL3	BANK1	CAMK2G
ACAA2	AGRN	ANG	ARHGAP18	AXL	C3	ACADVL	AKAP12	AP2M1	ATP1A2	BLVRA	CANX
ACAT2	AHNAK2	ANGPTL2	ARHGAP23	BDNF	C5orf24	ACO1	AKR1C1	APPL2	ATP2A1	BRWD1	CAPG
ACSL1	AHSG	ANXA2P1	ARHGEF1	BGN	C9orf19	ACSL4	AKR1C2	APRT	ATP2B3	BST1	CAPN1
ACTG2	AKR7A2	ANXA3	ARMS2	BHMT2	C9orf91	ACTBL2	ALPL	ARF6	ATP6V0D1	C20orf3	CAPN2
ACTN2	ALDH2	AP1S1	ARPC5	BRMS1	CACNA2D4	ADD1	ANKH	ARHGAP1	ATP6V1A	C3orf63	CAPN5
ACTN3	ALDH3A2	APEH	ASH1L	C11orf59	CAT	ADD3	ANXA2P2	ARPC1B	ATP6V1B2	CAB39	CBR1
ADAM9	ALDH6A1	APOA1	ASL	C1orf78	CCDC129	ADH5	AP1B1	ARPC2	ATP6V1E1	CALM1	CBR3
ADAMTS12	ALDH7A1	APOE	ATP8B3	C1R	CCDC64B	AHNAK	AP2A1	ASPH	AZGP1	CALML5	CCT2
CCL2	CEACAM8	CLEC11A	COL18A1	COPB1	CST4	CCT3	CLIC4	CSTA	DNAJC13	ECHS1	ENPP1
CCL20	CFB	CLIC6	COL2A1	COPS3	CTA-221G9.4	CCT4	CNP	CSTB	DPP3	EEF1A2	EPB41L1
CCL28	CFI	CLPX	COL3A1	COPS4	CTBP2	CCT7	COPA	CTSD	DPP4	EFEMP1	EPB41L2
CCL7	CFL2	CLSTN1	COL4A1	CREG1	CTNNA2	CCT8	COPB2	CUL4B	DPYSL3	EGFR	EPHX1
CCR4	CFTR	CLTA	COL4A2	CRIPT	CTSG	CDSN	CORO1C	CYB5R3	DSC1	EIF2S3	EPPK1
CCR5	CHMP2A	CLU	COL4A3	CRTAP	CXCL16	CEP290	COTL1	DAD1	DSG1	EIF3L	ERBB4
CD82	CHST12	CMIP	COL5A1	CSF1	CXCL2	CHI3L1	CPNE1	DDAH2	DSTN	EIF4A3	ERLIN2
CDC2L5	CITED1	CNGB1	COL5A2	CSF2	CXorf39	CHP	CPNE3	DDB1	DYNC1H1	EIF5A	ESD
CDIPT	CLASP2	COL12A1	COL7A1	CSF3	DBF4B	CKAP4	CRIP2	DDOST	DYNLL1	EMP3	ESYT1
CDK5R2	CLDN1	COL14A1	COMP	CSPG4	DCHS2	CLCA2	CSRP1	DIP2C	EBF3	ENDOD1	ESYT2
DCLK2	DNPEP	ENTPD4	FADD	FBLN1	FGF19	EZR	G6PD	GLO1	GPNMB	HSD17B12	IPO5
DCN	DPYS	ENTPD4;LOXL2	FAH	FBN1	FGFRL1	FABP5	GAB2	GNA11	GPX4	HSP90AA2	IPO7
DCTN1	DULLARD	EPB41L3	FAM108A1	FBN2	FGG	FAM129A	GAP43	GNA14	GSTM3	HSPA4	IQSEC1
DECR1	ECM1	EPHA2	FAM29A	FBXW8	FLJ13197	FAM26E	GBE1	GNAI1	GSTM4	HSPA9	ITGA1
DIP2B	ED1	EPO	FAM3B	FEN1	FLJ22184	FERMT2	GCN1L1	GNAI3	HADHA	HSPB6	ITGA10

DIRAS2	EDG2	ESM1	FAM64A	FER1L3	FLJ32784
DKFZp686D0972	EEA1	ETFB	FAM71F1	FGA	FLT1
DKK1	EFEMP2	F2R	FAT	FGB	FREM3
DKK3	EMILIN1	F3	FAT2	FGF16	FST
DMBT1	ENO3	F8	FAT4	FGF18	FTL

FUCA2	GFRA3	GRM3	HBB	HIST1H4H	HSPD1
GALNT5	GLDC	GRM7	HBE1	HIST2H2BE	HSPG2
GAPDHS	GLUD1	GSTM1	HDAC5	HLAA	HYI
GAS6	GNAT3	GSTM2	HERC5	HMGCS2	ICAM5
GDF1	GOT2	GSTM5	HGF	HNRNPA1	IDH3B
GDF11	GPC1	GTPBP2	HGFR	HP	IFNG
GDF3	GPC5	GYLTL1B	HISPPD2A	HPX	IFRD1
GDF5	GPR112	GZMA	HIST1H2AE	HRSP12	IFT140
GDF8	GREM1	H2AFV	HIST1H2BA	HSPA1L	IGFBP3
GDF9	GRM2	H2AFX	HIST1H2BL	HSPA6	IGFBP4

IGFBP6	IGKV1-5	IL1F9	IL8	KIAA0256	KRT73
IGFBP7	IGL@	IL1RAP	INHBA	KIAA0467	KRT74
IGHA1	IGLV4-3	IL1RAPL1	INHBB	KIAA1881	KRT79
IGHA2	IL10	IL1RL2	INSR	KRT15	KRT8
IGHG1	IL11	IL22RA1	ITGA4	KRT18	KRT80
IGHG2	IL13	IL23A	ITGAL	KRT19	LACRT
IGHG4	IL15RA	IL3	ITIH2	KRT27	LAMA4
IGHM	IL17B	IL5	ITIH4	KRT28	LAMB1
IGJ	IL17R	IL6ST	ITPR2	KRT7	LAMC1
IGKC	IL19	IL7	KIAA0146	KRT72	LCN1

LCN2	LOC284297	LRRFIP2	MARCKSL1	MMP1	NEFH
LDHAL6B	LOC388344	LTBP1	MAT1A	MMP10	NEK10
LEPRE1	LOC389827	LTBP2	MBD3	MMP3	NID1
LGALS3BP	LOC442497	LTF	MCC	MOS	NLRP8
LGALS8	LOC653269	LYAR	MCM10	MPO	NOMO1

FHL1	GFPT1	GNAQ	HEBP1	IDE	ITGA7
FHL2	GGT5	GNAT2	HLA-DRA	IDH1	ITGB3
FLG	GIPC1	GNB2L1	HLA-DRB1	IGFN1	JAK1
FSCN1	GJA1	GNPNAT1	HLA-H	ILK	KCTD12
FTH1	GLIPR2	GOLGA7	HRNR	IMPDH2	KIAA1609

KIF5B	KRT85	LRRN4CL	MOXD1	MYO6	NNMT
KPRP	KRTAP16-1	LTA4H	MTAP	MYOF	NOTCH2
KRT12	KRTAP2-1	MAP1B	MTHFD1	NAGK	NPC1
KRT24	KRTAP9-1	MAP4K4	MVP	NAPA	NPEPPS
KRT31	LASP1	MAPK1	MYL12B	NCEH1	NPTN
KRT33A	LCE3C	MAT2A	MYL6	NCKAP1	NQO1
KRT33B	LCP1	MLEC	MYL9	NCSTN	NSF
KRT71	LGALS7	MLL3	MYO1A	NDC80	NUDC
KRT75	LPAR1	MME	MYO1B	NDRG1	ODZ4
KRT81	LPHN2	MMP14	MYO1D	NFE2L3	OTUB1

PA2G4	PFKL	PLCD1	PRDX4	PRSS3	RAB13
PAFAH1B1	PFKM	PLEC	PRDX5	PSMD1	RAB18
PARK7	PFKP	PLIN3	PREP	PSMD10	RAB21
PCBP1	PGLS	PLS1	PRKACA	PSMD2	RAB22A
PCBP2	PGM1	PLS3	PRKAR2A	PSMD3	RAB23
PCMT1	PGRMC2	PLXND1	PRKCB	PTGES3	RAB31
PCYOX1	PHGDH	POTEE	PRKCDBP	PTGR1	RAB34
PDIA4	PKLR	PPAP2B	PRKCSH	PTPRA	RALB
PDIA6	PKP1	PPP2R1A	PRPH	PYGB	RAP1B
PDLIM5	PLAUR	PRDX2	PRSS1	PYGL	RAP2B

RDX	RPS13	S100A10	SEC23A	SND1	STX4
RECK	RPS14	S100A16	SELENBP1	SNTB2	STXBP1
RHOB	RPS17	S100A6	SEPT11	SNX3	STXBP3
RHOG	RPS25	S100A9	SEPT9	SNX9	SUSD2

LGR6	LOC727942	MADH4	ME1	MPZL1	NRG2
LIF	LOC728320	MAMDC2	MECP2	MXRA5	NRLN1
LMNA	LOC728378	MAP1A	MFAP4	MYCBPAP	NTF5
LOC124220	LOC730013	MAP2K6	MFSD2	MYH14	NUSAP1
LOC283523	LRP6	MAP3K1	MIF	NBL1	OBFC1
OFD1	PARP16	PLAB	PODN	PSMB5	PXDN
OPRM1	PARVG	PLAU	POLN	PSMB6	PZP
OSM	PC	PLEC1	POTE2	PSMB7	QPCTL
OTC	PDCD6	PLEKHG3	PPME1	PSMB8	QSOX1
OXNAD1	PDGFA	PLOD1	PRDM16	PSMB9	RAB15
OXTR	PDGFC	PLOD2	PRNP	PSMD14	RAB1B
PAICS	PFAS	PLOD3	PRR4	PTK7	RAB33B
PAN3	PFKFB3	PLTP	PRSS23	PTPRK	RAB39B
PAPPA	PGLYRP2	PLUNC	PSMA4	PTTG1IP	RAB6A
PARP10	PIGR	PNO1	PSMB10	PTX3	RAD21
RALA	RNF40	S100P	SEMA5A	SLAIN1	SLC7A10
RARRES1	RPL10A	SAA4	SERINC5	SLC1A4	SMARCA4
RASA1	RPL15	SASS6	SERPINA1	SLC22A2	SMC1A
RASA4	RPL29	SCAMP3	SERPINB3	SLC25A10	SORT1
RB1CC1	RPL35A	SCGB2A1	SERPINE1	SLC25A13	SPACA1
RCOR2	RPS10	SCYE1	SERPINF1	SLC2A1	SPARC
RDH5	RPS2	SDC1	SFN	SLC2A3	SPOCK1
RGN	RPS24	SDC2	SFRP1	SLC38A2	SPRY4
RMND5A	RPSA	SDC4	SFRP4	SLC38A3	SPTBN4
RNF123	RUVBL1	SEC14L4	SHANK3	SLC39A14	SRGN
SRI	SYT1	THBS1	TNC	TRIM40	TUBA1A
SRPX2	SYT9	THBS2	TNFRSF11B	TRIM41	TUBA1B
ST6GALNAC6	TAAR2	TIMP1	TNFRSF12A	TSNAX	UBA52
STC1	TAS2R60	TIMP2	TNFRSF1A	TSPAN14	UBB
STC2	TCN1	TMBIM1	TNFSF18	TSPAN4	UBE1

RNH1	RPS27	SAR1A	SERPINB12	SOD1	TAGLN2
RPL5	RPS3A	SAR1B	SERPINB6	SOD2	TARS
RPL7	RPS9	SBSN	SERPINH1	SQSTM1	TCP1
RPLP2	RRAS	SCARB2	SH3BGRL3	STEAP3	TMEM119
RPN1	RTN3	SDF2L1	SH3GLB1	STEAP4	TPM1
RPS11	RUVBL2	SEC22B	SNAP23	STIP1	TPM3
TPM4	UGDH	WARS	VPS26A		
TPP2	USP14	WDR1	VPS35		
TTBK2	USP5	XPNPEP1	UCHL1		
TUBA3C	USP9X	XPO1			
TUBA4A	VARS	YES1			
TUBB1	VAV2	YWHAH			
TXN	VDAC1	ZCCHC11			
TXNDC5	VDAC2	TXNRD1			

STOML3	TF	TMED9	TNFSF5	TSPAN6	UBE2N	
STX12	TFG	TMEM16B	TPBG	TSPAN9	UNC45A	
STX2	TGFB1	TMEM2	TRAP1	TSTA3	VANGL1	
SURF4	TGFB2	TMEM47	TREM1	TTLL3	VASN	
SVEP1	TGOLN2	TMEM51	TREML2P	TTYH3	VCAN	
VEGFC	WNT5A	WDR49				
VIL1	YBX1	WDR52				
VIL2	ZBTB4	ZNF614				
VTI1A	ZNF134					
VTN	ZNF503					

Supplementary data table 2. List of proteins which were identified in both articles, Kim et al., 2011 and Lai et al., 2012

**Common proteins list**														
ACLY	AHCY	ANXA2	ARPC4	CALR	CD151	CDH13	COPS8	DSP	ENO1	FLOT2	GNAS	HSP90AA1	IGF2R	JUP
ACTA1	AKR1B1	ANXA4	ATP1A1	CAND1	CD248	CFL1	CPS1	EDIL3	ENO2	FN1	GNB1	HSP90AB1	IGSF8	KPNB1
ACTA2	ALB	ANXA5	ATP1B3	CAP1	CD276	CLIC1	CTNNA1	EEF1A1	FAM129B	GANAB	GNB2	HSP90B1	IQGAP1	KRT1
ACTB	ALCAM	ANXA6	ATP2B1	CAPNS1	CD44	CLTC	CTNNB1	EEF1G	FAP	GAPDH	GNB4	HSPA1A	ITGA11	KRT10
ACTN1	ALDH9A1	ANXA7	ATP2B4	CAPZA1	CD47	CLTCL1	CTNND1	EEF2	FASN	GARS	GNG12	HSPA5	ITGA2	KRT13
ACTN4	ALDOA	ARF1	ATP5A1	CASP14	CD59	COL1A1	CYBRD1	EHD1	FLG2	GDI1	GNPDA1	HSPA8	ITGA3	KRT14
ACTR1A	ALDOC	ARF4	ATP5B	CAV1	CD63	COL1A2	DCD	EHD2	FLNA	GDI2	GPI	HSPB1	ITGA5	KRT16
ACTR2	ANPEP	ARHGDIA	BASP1	CCT5	CD81	COL6A1	DEFA1	EHD4	FLNB	GNA13	GSN	HTRA1	ITGAV	KRT17
ACTR3	ANXA1	ARL6IP5	BSG	CCT6A	CD9	COL6A2	DNASE1L1	EIF4A1	FLNC	GNAI2	GSTO1	ICAM1	ITGB1	KRT2
ADAM10	ANXA11	ARPC3	CACNA2D1	CD109	CDC42	COL6A3	DPYSL2	ENG	FLOT1	GNAL	GSTP1	IFITM2	ITGB5	KRT3
KRT4	LAMP1	MDH1	NME1	PEBP1	POSTN	PSMA6	PTGFRN	RAB5C	RHOC	RPS5	SLC16A1	STOM	TMED10	UGP2
KRT5	LAMP2	MDH2	NRAS	PFN1	PPIA	PSMA7	PTRF	RAB7A	RPL12	RRAS2	SLC16A3	TAGLN	TPI1	UNC13B
KRT6A	LAP3	MFGE8	NRP1	PFN2	PPIB	PSMB1	RAB10	RAB8A	RPL18	RTN4	SLC1A5	TALDO1	TSN	VAMP3
KRT6B	LDHA	MMP2	NT5E	PGAM2	PPP1CC	PSMB2	RAB11B	RAB8B	RPL23	S100A11	SLC3A2	TFRC	TTN	VAT1
KRT6C	LDHB	MRC2	ODZ3	PGD	PRDX1	PSMB3	RAB14	RAC1	RPLP0	S100A13	SLC44A1	TGFBI	TUBA1C	VCL
KRT76	LGALS1	MSN	P4HB	PGK1	PRDX6	PSMB4	RAB1A	RAC2	RPS16	S100A8	SLC44A2	TGM2	TUBB	VCP
KRT77	LGALS3	MYADM	PCOLCE	PIP	PSMA1	PSMC5	RAB2A	RAN	RPS18	SDCBP	SLC7A5	THY1	TUBB2A	VIM
KRT78	LRP1	MYH9	PDCD6IP	PKM2	PSMA2	PSMD11	RAB35	RAP1A	RPS27A	SEPT2	SPTAN1	TIMP3	TUBB2C	YWHAB
KRT84	LYZ	MYL6B	PDGFRB	PLP2	PSMA3	PSMD6	RAB5A	RAP2C	RPS3	SEPT7	SPTBN1	TKT	TUBB3	YWHAE
KRT9	MARCKS	MYO1C	PDIA3	PLSCR3	PSMA5	PSMD7	RAB5B	RFTN1	RPS4X	SERPINE2	STAT1	TLN1	TUBB6	YWHAG
YWHAQ	YWHAZ													
